# Near-Infrared Spectroscopy and Imaging Studies of Fertilized Fish Eggs: *In Vivo* Monitoring of Egg Growth at the Molecular Level

**DOI:** 10.1038/srep20066

**Published:** 2016-01-28

**Authors:** Mika Ishigaki, Shoya Kawasaki, Daitaro Ishikawa, Yukihiro Ozaki

**Affiliations:** 1 School of Science and Technology, Kwansei Gakuin University, 2-1 Gakuen, Sanda, Hyogo 669-1337, Japan; 2 Graduate School of Agricultural Science, Tohoku University, 1-1 Amamiya, Tsutsumidori, Aobaku, Sendai 981-8555, Japan

## Abstract

In this work, the growth of fertilized Japanese medaka (*Oryzias latipes*) eggs was monitored *in vivo* at the molecular level using near-infrared (NIR) spectroscopy and NIR imaging. NIR spectra were recorded noninvasively for three major parts of a fertilized medaka egg, the embryonic body, the oil droplets, and the yolk, from the first day after fertilization to the day before hatching. Principal component analysis (PCA) revealed that water, protein, and lipid contents in the egg yolk and oil droplets changed significantly just before hatching. The ratio of the characteristic peaks due to proteins and lipids in the second derivative spectra suggested that the relative concentration of proteins to lipids was constant in the egg yolk, while it dramatically increased just before hatching in the oil droplets. Furthermore, linear discriminant analysis (LDA) predicted the hatching possibility on the next day with 100% and 99.3% accuracy for yolk and oil droplets data, respectively. Two types of NIR images were developed *in situ* using the band intensities of the lipids and proteins in the second derivative spectra. The egg’s protein and lipid content was successfully visualized noninvasively. This technique should enable noninvasive quality testing of fertilized eggs in the future.

“Embryonic quality” is a key consideration for marine culture, the livestock industry, and fertilization treatments for humans. Embryonic quality has been shown to have a close relationship with the survival potential of an embryo. Many studies on marine culture have investigated the estimation of embryonic quality from the aspect of the stabilization of fishery resources, reproduction of wild fish, and preservation of endangered species, and the factors affecting egg quality have been reported^1^,^2^,^3^,^4^,^5^,^6^. For example, the egg of sea bass (*Dicentrarchus labrax* L.) can be assessed by observing whether it floats or sinks in seawater^2^,^3^. However, this method to assess the egg quality cannot be applicable universally to all species. There are also other studies that have examined the relationship between the egg quality and dietary fatty acids^1^,^7^.

Furthermore, in human fertility treatments, it is well known that embryonic quality assessed by a grading method based on cleavage rates and the morphological features of the ovum is closely related to pregnancy rates after *in vitro* fertilization (IVF)^8^,^9^,^10^,^11^. The grading method of embryonic quality is actually used in clinical practice of IVF. However, the method seems undeniably to be empirical and phenomenalistic, and it does not declare what the embryonic quality is. Therefore, it is desired that new, nondestructive evaluation techniques of embryonic quality that monitor chemical changes of the inner components via spectroscopic analysis will contribute to improving the success rates of IVF treatments. In this study, ability to monitor changes in the relative concentrations of chemical components in eggs such as proteins and lipids was demonstrated during embryonic development.

An egg from the Japanese medaka (*Oryzias latipes*) was suitable for the analysis of embryonic development. This fish is easy to cultivate and is often used as a laboratory model to test compounds for carcinogenicity^12^,^13^. Furthermore, the egg of this species is amenable to spectroscopic analysis, because it is transparent. A normal Japanese medaka egg hatches approximately 2 weeks after fertilization under usual feeding conditions^14^,^15^. In the unfertilized egg, a number of small oil droplets are visible in the egg. Soon after fertilization, the oil droplets begin to coalesce and fuse into bigger droplets. In regions where there are no large oil droplets, cytoplasm gathers to form a blastodisc, which transform into the embryonic body. The oil droplets are rich in lipids (unsaturated fatty acids), while egg yolk contains both proteins and lipids^15^,^16^. An anomalous egg, on the other hand, halts development, and its ontogenesis stops.

It is of particular importance for both basic science and practical applications that egg growth is observed *in vivo* at the molecular level. If the relative contents of lipids and proteins could be estimated over time in the oil droplets, egg yolk, and embryonic body, growth anomalies could be detected. In other words, egg quality could be monitored *in situ*.

Near infrared (NIR) spectroscopy is a vibrational spectroscopy like infrared (IR) and Raman spectroscopies^17^,^18^. When a light in a wavelength region is irradiated on molecules, the transition of energy level takes place corresponding to the vibrational transition energy. In NIR spectroscopy, overtone and combination modes of the vibrational energy transitions, so-called forbidden transitional mode, can be detected. The absorbance of these modes is very weak compared to the one of allowed transitional modes in IR region. For example, one can only measure samples with high water content in the order of micrometer thickness within mid-IR region, while in the NIR region, the order of millimeter. NIR light is superior in terms of permeability and NIR spectroscopy is more applicable to non-destructive and *in situ* analysis. Furthermore as the experimental fact, NIR spectra could be obtained without any problems compared to Raman spectroscopy in which sensitivity of Raman spectra got low because of the auto-fluorescent with the egg developments.

In recent years, NIR spectroscopy and NIR imaging have made marked progress in instrumentation, spectral analysis, and applications^17^,^18^,^19^,^20^,^21^,^22^,^23^. One of the most attractive advantages of NIR spectroscopy is its ability to provide molecular information nondestructively. NIR spectroscopy is useful not only for molecular structural studies of hydrogen bonds but also for the nondestructive analysis of various materials such as foods, tablets, and polymers^17^,^18^,^19^,^20^,^21^,^22^,^23^. In the application of NIR spectroscopy to medicine, many researches have been reported about brain function^24^,^25^,^26^, cancer^27^,^28^, skin^29^,^30^, and diabetes^31^,^32^.

NIR imaging has recently received much interest, because it provides the spatial distribution of chemical components nondestructively even for thick materials. Many studies such as quality evaluations of pharmaceutical tablets, dissolution processes of tablets and polymers, and the component and crystallinity distributions of polymers were reported^33^,^34^,^35^,^36^,^37^,^38^,^39^. In the application of NIR imaging to biology and medicine, some papers examined skin hydration^40^,^41^, human cancer tissues^42^, were already published. NIR imaging studies on human brain function, which are based on NIR electronic spectroscopy, have been very active^43^,^44^. But NIR imaging is also infrequently used to study problems of basic biology.

The purpose of the present study is to explore the growth process of fertilized eggs from Japanese medaka noninvasively using NIR spectroscopy and NIR imaging. Variations in an egg’s protein and lipid contents were investigated *in situ* in the embryonic body, egg yolk, and oil droplets separately. Changes in chemical distributions were visualized using NIR imaging. Principal component analysis (PCA) discriminated the growth of fertilized eggs in terms of the relative concentration change of proteins. And linear discriminant analysis (LDA) discriminated the hatching possibility of the fish egg with water, lipid, and protein bands. The present study may provide a new molecular tool for visualizing egg growth.

## Materials and Methods

### Breed of Japanese medaka (*Oryzias latipes*)

Medaka was bred in a small fish tank at 25 °C. They were fed a commercial Medaka fish food. The eggs produced were approximately 1.5 mm in diameter. After fertilization, the eggs began embryonic development. The detailed processes of this development are explained in ref. [14].

**Figure 1:**
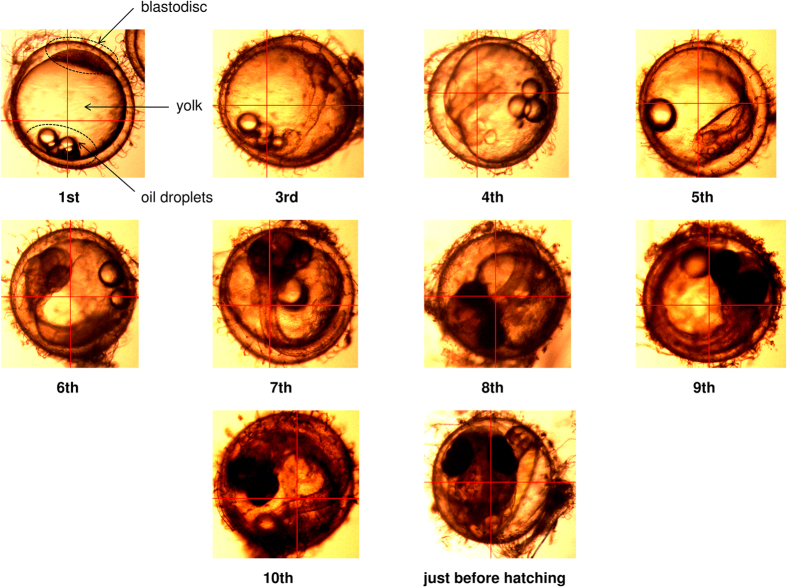
Photos showing the growth process of a fertilized medaka egg from one day after fertilization to the day before hatching taken with a microscope at ×5 power.

Figure 1 shows photographs of the growth process of a fertilized medaka egg from the first day after fertilization to the day before hatching. The egg consists of three major parts, the oil droplets, egg yolk, and blastodisc, as shown in Fig. 1. Unequal cleavage takes place at the blastodisc during egg development, and the blastodisc is transformed into an embryonic body. After 6^th^ day after fertilization, the embryonic body can be easily seen in the egg as shown in Fig. 1. The egg contains sufficient energy to support the egg’s development until first feeding^45^. Much energy is contained within the oil droplets and egg yolk, and total lipid content was estimated to decrease 30% during the egg development^46^.

The eggs of Japanese medaka hatch about 2 weeks after fertilization, but the term before hatching is variable. In our study, the eggs hatched in 11 to 14 days. In most cases, the eggs spawned by one individual hatched on the same day. Now we consider the case in that one egg spawned by one individual was picked up and NIR measurement was performed. If the other eggs spawned by the same individual hatched on the next day after the NIR measurement, the NIR data were assigned as “just before hatching”.

When NIR spectra were obtained in single point mode, three eggs were selected at each developmental stage from the first day after fertilization to the day before hatching as shown in Fig. 1. Ten points for each three part of the egg, the oil droplets, egg yolk, and blastodisc, were measured in order to reduce individual variability and dependence on measurement point. After the measurement, the averaged NIR spectra were calculated for each day and each part. The data on the 2^nd^ day were not obtained because the border between blastodisc and egg yolk temporarily became unclear.

**Figure 2:**
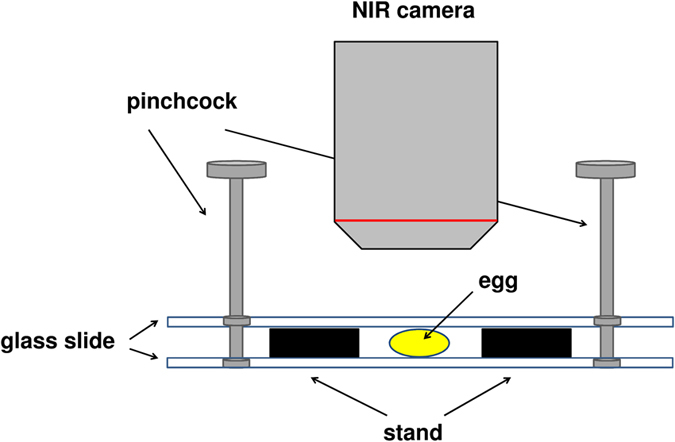
Schematic view of the egg measurement by NIR spectroscopy and NIR imaging.

The eggs were almost spherical in form. To regulate the optical path length, NIR measurements were performed by sandwiching the egg between two glass slides with pinchcocks as shown in Fig. 2. The thickness of the egg became about 0.36 mm, whereas the original egg size was 1.5 mm.

We performed the experiments in accordance with the fundamental guidelines for proper conduct of animal experiment and related activities in academic research institutions under the jurisdiction of Ministry of Education, Culture, Sports, Science and Technology in Japan. The study was approved by the ethics committee of Kwansei Gakuin University.

### Instrumentation and NIR measurements

The Fourier transform near-infrared (FT-NIR) spectral measurements and imaging measurements were performed using a Perkin-Elmer (Waltham, MA) imaging system consisting of a Spectrum One FT-NIR spectrometer coupled to a Spectrum Spotlight 300 NIR microscope. This integrated instrument includes a 16 element (400 × 25 μm^2^) HgCdTe (MCT) array detector and a single-point 100 × 100 μm^2^ MCT detector in the same Dewar. The symmetrically arranged objective and condenser provided an image magnification of 6× at 1:1 imaging and a numerical aperture of 0.58. Specifically designed optics permitted 1:1 or 4:1 imaging of the sampled area on the detector elements, resulting in 25 × 25 μm^2^ or 6.25 × 6.25 μm^2^ pixel sizes. For the present investigations each image was measured in the transmission mode over an area of 1.5 × 1.5 mm^2^ with a pixel size of 25 × 25 μm^2^ and a spectral resolution of 8 cm^−1^ by coadding 8 scans for a spectrum in the 6200–4000 cm^−1^ region.

In order to extract the practical information about the variation factors with the egg development from NIR data, multivariate analyses such as PCA and LDA were performed. In our study, they were performed with chemometrics software Unscrambler (CAMO, USA).

## Results and Discussion

**Figure 3:**
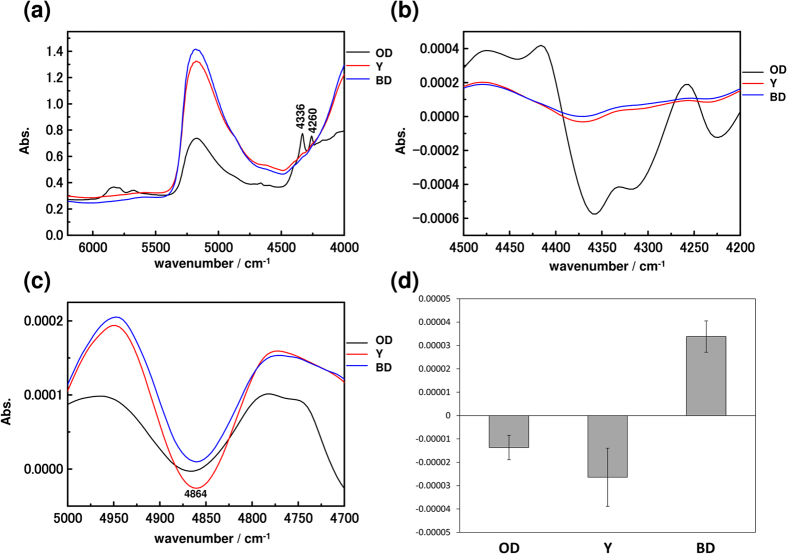
(**a**) NIR spectra in the 6200–4000 cm^−1^ region of the blastodisc, oil droplets, and egg yolk on the fertilization day (1st day). (**b**,**c**) Second derivative spectra in the 4500–4200 cm^−1^ and 5000–4700 cm^−1^ region, respectively. (**d**) A graph of the average intensity of the second derivative of proteins at 4864 cm^−1^ ± 1SE (standard error). (OD: oil droplets, Y: yolk, BD: blastodisc).

Figure 3(a) compares the NIR spectra of three major parts of the fertilized egg on the first day. All of the spectra show a broad feature near 5200 cm^−1^ due to the combination of the antisymmetric OH stretching mode and the bending mode of water. The spectra of oil droplets show several strong bands attributed to lipids. Figure 3(b,c) show second derivative spectra in the 4500–4200 cm^−1^ and 5000–4700 cm^−1^ regions, respectively. Two intense bands near 4260 and 4336 cm^−1^, which can be assigned to the combination of CH stretching and bending modes of lipids, and weak features at 4864 cm^−1^, which are due to a combination of the NH stretching mode and the amide II mode of egg proteins, were observed^17^,^18^. According to Fig. 3(b), fatty acids are predominantly in the oil droplets. On the other hand, the concentration distribution of proteins cannot be determined based on the data in Fig. 3(c,d) shows a graph of the average intensity of the second derivative at 4864 cm^−1^. A high concentration of proteins was confirmed in the yolk.

**Figure 4:**
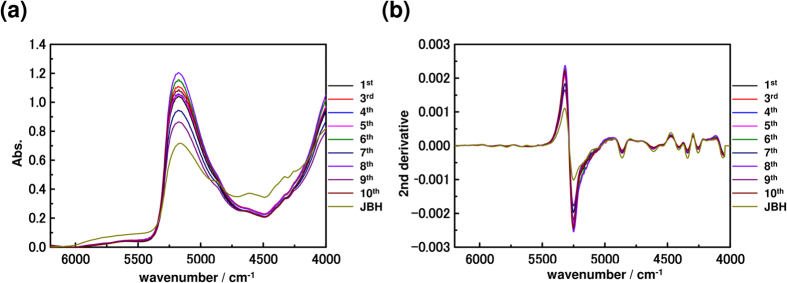
(**a**) NIR spectra, preprocessed with baseline correction, in the 6200–4000 cm^−1^ region and (**b**) their second derivatives of the yolk from the first day after fertilization to the day before hatching. (JBH: just before hatching).

**Figure 5:**
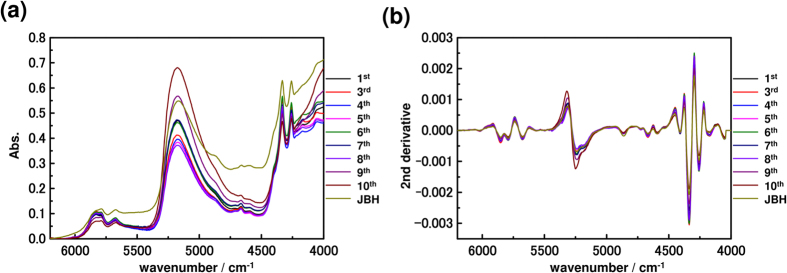
(**a**) Second derivative spectra with baseline correction in the 6200–4000 cm^−1^ region and (**b**) second derivatives of the oil droplets from the first day after fertilization to the day before hatching. (JBH: just before hatching).

Figures 4 and 5 show NIR spectra and their second derivatives of the yolk and oil droplets, respectively. It should be noted that the shape of the water band around 5252 cm^−1^ is significantly different in the second derivative spectra of the yolk and of the oil droplets. The relative intensity at 5250 cm^−1^ due to free water (weakly hydrogen bonded water) is very intense in the egg yolk spectra^47^,^48^.

**Figure 6:**
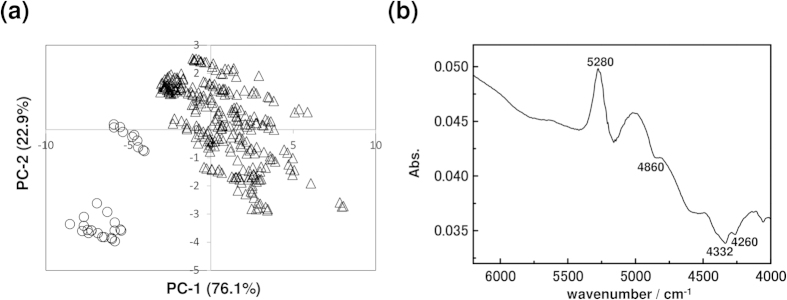
(**a**) PC-1 and PC-2 PCA score plot of all NIR spectra of yolk measured in the period from day one to the day before hatching. △ is data from the 1^st^ to 10^th^ day and ○ is data from the day before hatching. (**b**) Loading plot of PC-1.

In order to extract variations in the components during egg development, PCA was performed on the data set measured over the course of the egg’s development. Figure 6(a) shows principal component 1 (PC-1) and principal component 2 (PC-2) score plots of all of the NIR spectra of yolk. The spectra of the yolk just before hatching were clearly separated from the others. The loading plot of PC-1 demonstrated the separation factor of the data set into two groups, one from the first to tenth day after fertilization and the other for just before hatching, and had peaks attributed to the combination of antisymmetric OH stretching mode and bending mode of water (5280 cm^−1^), a combination of the NH stretching mode and amide II mode of proteins (4860 cm^−1^), and the combination of CH stretching and bending mode of lipids (4260, 4332 cm^−1^)^17^,^18^. In other words, these components changed significantly just before hatching. Furthermore, the peak directions of proteins and lipids were the same.

**Figure 7:**
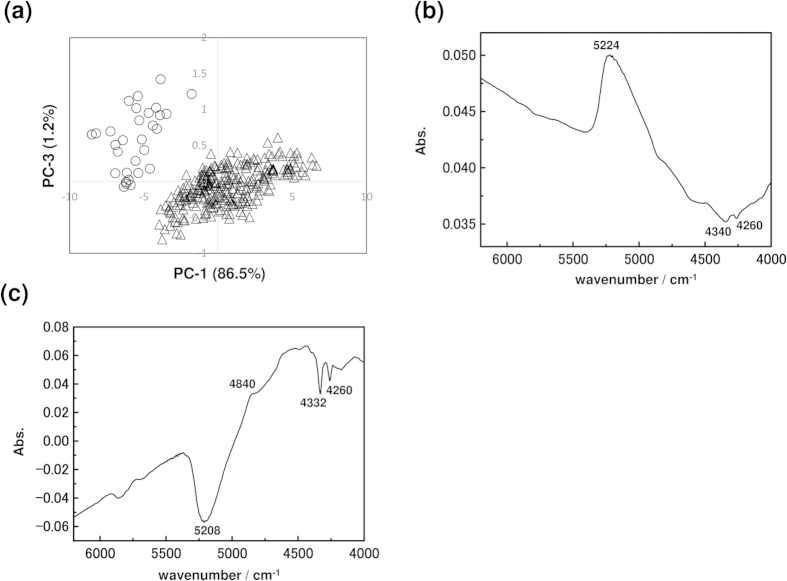
(**a**) PC-1 and PC-3 PCA score plot of all NIR spectra of lipid measured from day one to the day before hatching. △ is data from 1^st^ to 10^th^ day and ○ is data from the day before hatching. (**b**,**c**) Loading plots of PC-1 and PC-3, respectively.

PCA results for the lipid data set shows a similar classification pattern between a group just before hatching and a group containing all other days as shown in Fig. 7. Peaks due to water, proteins, and lipids also appear in the loading plots of PC-1 and PC-3. However, the directions of protein and lipid peaks are opposite.

**Figure 8:**
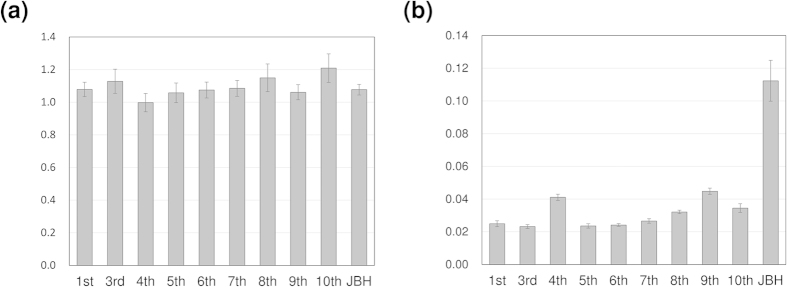
Intensity ratio ±1SE (standard error) versus day after fertilization. The relative content of lipids and proteins is calculated by the relative intensity of the two characteristic peaks at 4864 cm^−1^ (proteins) and 4336 cm^−1^ (lipids) in the second derivative spectra of the (**a**) yolk and (**b**) oil droplets.

In order to compare the relative variations of these components, the ratio of the two characteristic peaks at 4864 cm^−1^ (proteins) and 4336 cm^−1^ (lipids) in the second derivative spectra of yolk and oil droplets were estimated. Figure 8 plots the intensity ratio as a function of day after fertilization. The intensity ratio in the yolk remains almost constant over time as shown in Fig. 8(a). Meanwhile in the oil droplets, the relative content of protein dramatically increased just before hatching by a factor of 3 to 4 as seen in Fig. 8(b). The results were consistent with the discussion about peak directions of proteins and lipids which appeared in the loading plot of the PCA of the yolk and oil droplets as mentioned above. When the egg is close to the hatching stage, the embryo secretes the hatching enzyme from the hatching gland cells^49^,^50^. The molecular weight of the enzymes of Medaka was estimated approximately as 8000 by HPLC analysis^51^. The NIR result that the relative content of protein in the oil droplets jumped up indicates the release of the hatching enzyme. Furthermore, fish grow after hatching with only oil components stored in their abdomens until their first feeding^45^. It was reported that only phosphatidylcholine was used during embryogenesis and the energy source shifted to neutral lipid after hatching^52^. The variations of protein and lipid components suggested by PCA may be caused by changes in the energetic metabolism near hatching. The result indicates that NIR spectroscopy can be used to monitor metabolic changes that occur just before hatching.

**Figure 9:**
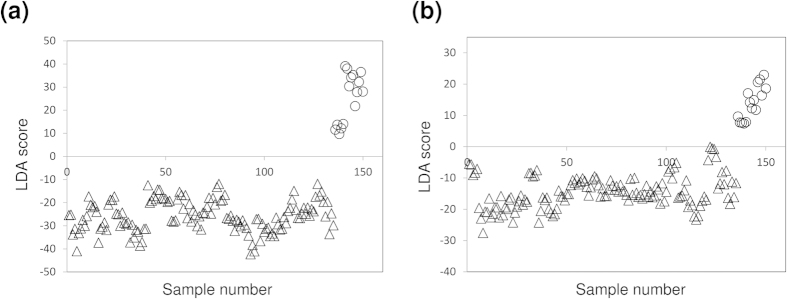
Score plot of LDA validation. △ expresses an egg from 1^st^ to 10^th^ day after fertilization and ◦ is an egg just before hatching. 150 samples were used to create a LDA model and remaining 150 samples were validated. (**a**,**b**) show the validation results of yolk and lipid, respectively.

Next, LDA was performed to confirm whether NIR spectroscopy can predict the hatching possibility of the egg on the next day. The LDA model was obtained by using water (5250 cm^−1^), lipid (4260, 4332 cm^−1^) and protein (4860 cm^−1^) bands. The number of each NIR data for yolk and oil droplets was 300. To develop the LDA model, 150 samples were used and the reliability of the model was validated. The hatching possibilities of all yolk samples were correctly predicted with 100% accuracy, whereas 149 samples out of 150 were correctly predicted and 1 were misjudged about the oil droplets data, with accuracy of 99.3% as shown in Fig. 9. That is, the eggs on the stage of just before hatching were significantly different from the eggs on the other stages and NIR spectroscopy can predict the hatching possibility of the egg.

The reproducibility of PCA and LDA were tested with the data set of more than double sample numbers. The new PCA results also showed that the data on the stage of just before hatching were separated from the data on the other developmental stages. Furthermore, new LDA successfully predicted the hatching possibility on the next day with more than 90% accuracy for yolk and oil droplets data set.

**Figure 10:**
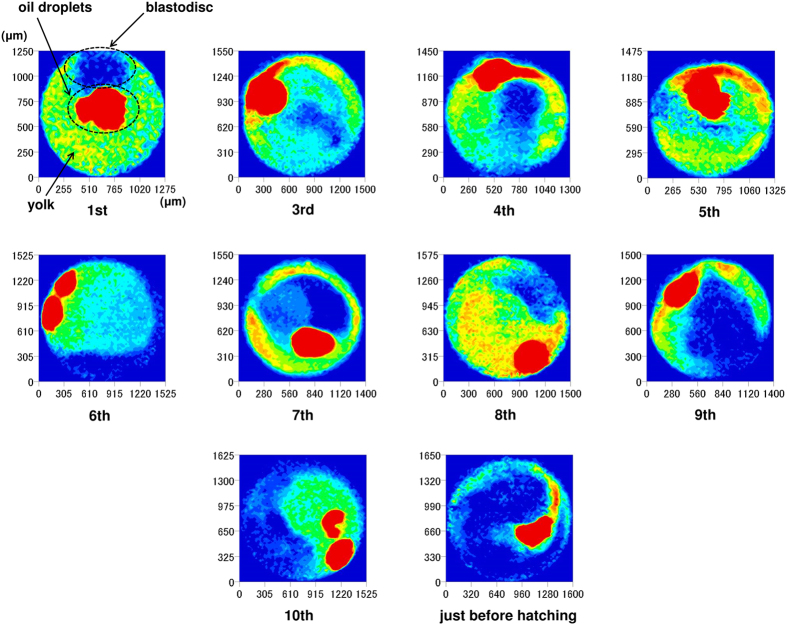
NIR images at 4336 cm^−1^ due to lipids in the second derivative spectra of fertilized medaka eggs.

**Figure 11:**
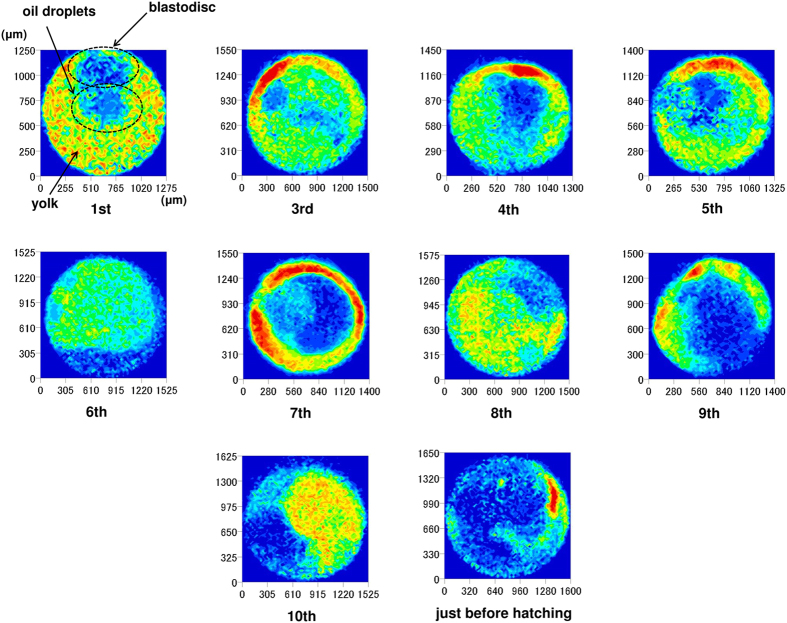
NIR images at 4864 cm^−1^ due to proteins in the second derivative spectra of fertilized medaka eggs.

Figure 10 shows NIR images taken at 4336 cm^−1^, showing lipids in the second derivative spectra of fertilized medaka eggs. The corresponding images taken at 4864 cm^−1^, which is due to proteins, are presented in Fig. 11. In both cases, day-dependent variations of protein and lipid distributions were observed in the fertilized medaka eggs between fertilization and hatching. This noninvasive method clearly shows that the lipid distribution changes with time. Lipids always concentrate in the oil droplets. Lipid content is also considerable in other parts of the egg in the first several days, but it becomes lower over time. The distribution of egg proteins is homogeneous in the first stages of development, but it becomes more heterogeneous each day. The variation, localization, and concentration gradient of the egg’s interior components were successfully visualized by NIR imaging.

## Conclusion

This study presented the *in vivo* monitoring of the growth of fertilized eggs of Japanese medaka at the molecular level using NIR spectra and imaging. NIR spectra in the 6200–4000 cm^−1^ region were measured noninvasively for three major parts of a fertilized medaka egg, the embryonic body, oil droplets, and egg yolk, over the time period from the first day after fertilization to the day before hatching.

PCA of the second derivative spectra showed that the egg underwent significant changes just before hatching. The protein and lipid components changed just before hatching. The ratio of the two characteristic peaks at 4864 cm^−1^ (proteins) and 4336 cm^−1^ (lipids) in the second derivative spectra suggested that the relative protein content in the oil droplets dramatically increased just before hatching, while the change was less pronounced in the egg yolk. These results indicate that the hatching enzymes secreted to digest the egg envelop or the energetic metabolism of oil components starts just before hatching to prepare for growth after hatching. NIR spectroscopy can decipher signs of hatching and metabolic changes in the egg noninvasively. Furthermore, LDA could discriminate hatching possibility on the next day by using water, protein and lipid bands, with 100% and 99.3% accuracy, for yolk and oil droplets data, respectively. The result indicates that the eggs on the stage of just before hatching are significantly different from other stages about these biological components.

NIR images were developed *in situ* using the intensities of the bands at 4336 cm^−1^, which is associated with lipids, and 4886 cm^−1^, which is associated with egg proteins, in the second derivative spectra. The variations in protein and lipid distributions in the eggs can clearly be monitored *in situ*. The present study demonstrates the potential of NIR spectroscopy and NIR imaging for the *in-situ* monitoring of the growth of various types of fertilized eggs for both basic studies and practical applications.

## Additional Information

**How to cite this article**: Ishigaki, M. *et al*. Near-Infrared Spectroscopy and Imaging Studies of Fertilized Fish Eggs: *In Vivo* Monitoring of Egg Growth at the Molecular Level. *Sci. Rep.* **6**, 20066; doi: 10.1038/srep20066 (2016).
